# Evaluation of Inulin and Polyphenol Content and the Cytotoxicity of *Cichorium intybus* L. var. *foliosum* Root Extracts Obtained by Pectinase- and Pressure-Assisted Extraction

**DOI:** 10.3390/nu17061040

**Published:** 2025-03-16

**Authors:** Łukasz Duda, Grażyna Budryn, Monika Anna Olszewska, Magdalena Rutkowska, Weronika Kruczkowska, Katarzyna Grabowska, Damian Kołat, Andrzej Jaśkiewicz, Zbigniew Włodzimierz Pasieka, Karol Kamil Kłosiński

**Affiliations:** 1 Department of Biomedicine and Experimental Surgery, Faculty of Medicine, Medical University of Lodz, Narutowicza 60, 90-136 Lodz, Poland; weronika.kruczkowska@stud.umed.lodz.pl (W.K.); katarzyna.grabowska1@stud.umed.lodz.pl (K.G.); damian.kolat@umed.lodz.pl (D.K.); zbigniew.pasieka@umed.lodz.pl (Z.W.P.); 2 Institute of Food Technology and Analysis, Faculty of Biotechnology and Food Sciences, Lodz University of Technology, B. Stefanowskiego 2/22, 90-537 Lodz, Poland; grazyna.budryn@p.lodz.pl; 3 Department of Pharmacognosy, Faculty of Pharmacy, Medical University of Lodz, Muszynskiego 1, 90-151 Lodz, Poland; monika.olszewska@umed.lodz.pl (M.A.O.); magdalena.rutkowska@umed.lodz.pl (M.R.); 4 Department of Functional Genomics, Faculty of Medicine, Medical University of Lodz, Zeligowskiego 7/9, 90-752 Lodz, Poland; 5 Department of Sugar Industry and Food Safety Management, Faculty of Biotechnology and Food Science, Lodz University of Technology, Wolczanska 171/173, 90-530 Lodz, Poland; andrzej.jaskiewicz@p.lodz.pl; 6 Biomaterials Research Laboratory, Faculty of Medicine, Medical University of Lodz, Narutowicza 60, 90-136 Lodz, Poland

**Keywords:** chicory extracts, chicory root, cytotoxicity, inulin, polyphenols, phenolic acids

## Abstract

Background: *Cichorium intybus* L., a member of the Asteraceae family, has numerous health-promoting properties that categorize its preparations as functional foods and herbal medicines. Most previous research focused on the root of *C. intybus* var*. sativum* (industrial chicory) as a rich source of inulin, while the witloof variety (*C. intybus* var. *foliosum*) is less explored. Objectives: This study aimed to evaluate the cytotoxicity of *C. intybus* var. *foliosum* root extracts obtained with different extraction protocols and to analyze their polysaccharide and polyphenol content. Methods: Freeze-dried root extracts were prepared using water and three extraction methods: pectinase-assisted, pressure-assisted, and a combination of both. The contents of inulin, total polyphenols, and total caffeic acid derivatives in the extracts were measured by the Layne–Eynon, Folin–Ciocalteu, and UHPLC-PDA methods, respectively. Cytotoxicity of the extracts and inulin was tested in vitro using the L929 cell line, MTT method, and paracetamol as the reference standard. Results: Inulin levels in the extracts ranged from 43.88 to 50.95 g/100 g dry matter (dm), total polyphenols were between 816.7 and 906.4 mg/100 g dm, and total phenolic acids ranged from 11.50 to 187.1 mg/100 dm, with pressure-assisted extraction yielding the highest phytochemical recovery. The cytotoxicity tests showed IC_50_ values from 4.72 to 7.31 mg/mL for the extracts, compared to 3.02 for paracetamol and 19.77 for inulin. Conclusions: Given the high content of active compounds and low cytotoxicity, the root extracts of *C. intybus* var. *foliosum* merit further research into their functional and medicinal properties. Pressure-assisted extraction is recommended for effective extraction of chicory.

## 1. Introduction

*Cichorium intybus* L., commonly known as chicory, is a woody perennial herb from the Asteraceae family, growing up to 1 meter tall. It features a fleshy taproot about 70 cm long, and large leaves at the base [1]. The plant name combines Greek and Latin origins, with “*Cichorium*” meaning field and “*intybus*” relating to the Latin “*tubus*”, concerning stem structure, or “*to cut*” in Greek, regarding leaf morphology. Although chicory originates from Europe, it has been naturalized globally, including in Africa, Asia, Australia, and North and South America [2,3].

The chicory leaves are used as a vegetable, the powdered root as a coffee substitute, and the whole plant as animal feed. Historically, many civilizations, such as the ancient Egyptians and Romans, also utilized various chicory parts for medicinal purposes. Chicory flowers have been employed as a tonic, appetite stimulant, and in treating gastritis, intestinal inflammation, gallstones, skin problems, and sinuses, while whorl decoctions were used as laxatives [1]. Moreover, aqueous root preparations have served as anti-malarian agents [4]. Today, the primary medicinal applications of chicory concern mild digestive disorders, appetite loss, and liver diseases; however, its use in lung, prostate, and reproductive organ disorders, as well as cough, malaria, and cancer, is also reported [1,3].

**Figure 1:**
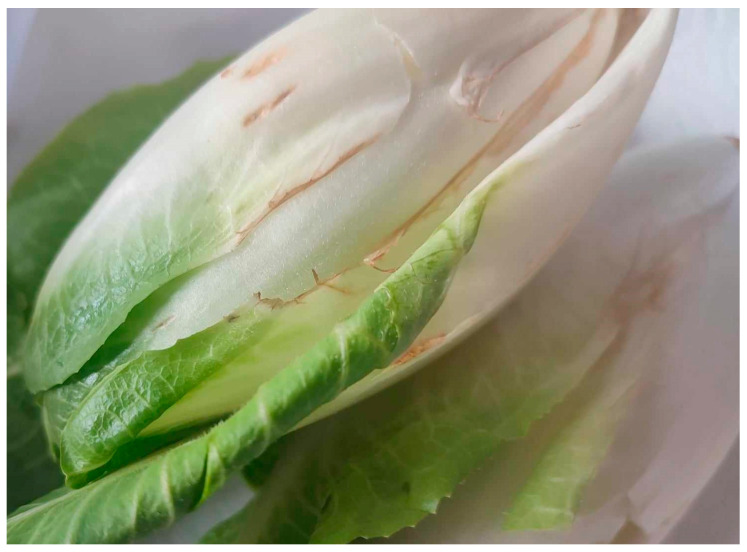
*Cichorium intybus* L. var. *foliosum* aerial parts (a vegetable).

Chicory is cultivated worldwide in two main varieties: *C. intybus* var*. sativum* (industrial chicory) for root production and inulin extraction, and *C. intybus* var. *foliosum* (witloof, Belgian endive) for cream-colored, bitter vegetable buds forced in darkness (Figure 1). Although the witloof root is less utilized, it recently gained increased attention as a potential health-promoting, inulin-containing product [5].

The main industrial value of chicory root is related to inulin—a reserve carbohydrate in chicory and a fructose polymer having β-2,1-bonds with the terminal glucose unit. As a polyfructan-containing agent, the root does not influence the blood sugar level; thus, it is suitable food for diabetic patients and diabetes prevention [6,7]. The total saccharide fraction of chicory root accounts for up to 60–80% of the dry weight of the plant material, with the inulin fraction making up 50 to 70% and the co-occurring simple sugars, e.g., glucose, fructose, and sucrose [8,9,10]. The sugar profile varies between chicory varieties, and cultivation and storage conditions [10,11,12].

Chicory also contains other phytochemicals that can be considered bioactive substances; these include mainly bitter sesquiterpene lactones, like germacranolides and guaianolides, with lactucin and lactucopicrin as primary structures; phenolic acids with caffeoyl pseudodepsides, primarily chlorogenic acids, as leading compounds; flavonoids with quercetin, kaempferol, and isorhamnetin as the most abundant aglycones; as well as sterols, polyacetylene, and volatiles in varying amounts [3,13]. The total polyphenol content in chicory is strongly organ-dependent and the highest in leaves (52–386 mg/100 g fresh weight, fw) [14]. In roots, the polyphenolic fraction accounts for up to 20–35 mg/100 g dm in the plant material and 0.2–7.9 mg/g dm in the extract, depending on the plant variety, cultivation conditions, and extraction solvent [10,15]. Similarly, the total sesquiterpene levels varied between different chicory organs and varieties, accounting for up to 0.1–2.4 mg/g dm in leaves and 0.1–0.4 mg/g dm in roots [10,15].

Accumulated knowledge indicates that *C. intybus* has many health-promoting properties that correlate to its application in traditional medicine. Phytotherapy is a living tradition that has found its place in contemporary medicine as a complementary therapy, a disease-preventing strategy, or for mitigating the effects of synthetic drug treatment [16]. In many regions, herbal medicines and “traditional” or handed-down treatments are still the main, and sometimes even the only, source of health care. To effectively employ the healing potential of traditional phytotherapy, efforts are being made in most WHO (World Health Organization) countries to confirm the safety and quality of herbal cures [11,17].

While chicory root is known mainly as a coffee substitute, it can also serve as a functional food additive [15,18,19]. Underpinning the benefits of chicory root is its ability to support intestinal health. Inulin acts as a nutrient for beneficial gut bacteria, supporting the development of the microbiome that, in turn, improves digestion and lowers inflammation and the chance of constipation. In addition to improving gut function, chicory root has shown promise in controlling blood sugar levels. Studies suggest that inulin may increase insulin sensitivity, thus making it a promising anti-diabetic agent. The high fiber content also contributes to feelings of satiety, aiding weight-management efforts [20,21]. In turn, antioxidants found in chicory root, primarily rich fraction of polyphenols such as chlorogenic acid, but also other constituents, protect the body from oxidative stress and inflammation [22,23]. They may play a preventive role against cardiovascular, cancer, or neurodegenerative disease development [11]. It is worth noting that the recent tests on simulated human gastrointestinal digestion in vitro revealed enhanced anti-inflammatory and antioxidant properties of chicory root resulting from the specific transformations of chlorogenic acid and sesquiterpene lactones during digestion. A significant increase in metabolite activity and ability to scavenge reactive oxygen species (ROS) was demonstrated [24]. These characteristics make chicory a promising functional food ingredient for improving overall well-being.

Chicory is currently being researched for its potential to treat various health disorders, from digestive problems and inflammation to viral infections. Early results suggest that the plant may also benefit cardiovascular health, blood sugar level, and immunity [1,15,25,26]. Notably, a study by Nishimura et al. found a decrease in hemoglobin A1c levels and increased adiponectin in participants consuming chicory root extract, indicating potential for delaying diabetes and improving defecation [21]. The controlled trial on pigs showed significant changes in liver cytoskeleton protein expression and cholesterol and triglyceride metabolism due to chicory root supplementation, which may be linked to its anti-inflammatory properties and downregulation of cytokeratin 18, an acute phase protein of the liver [27]. Another study demonstrated pro-apoptotic, anti-inflammatory, antimicrobial, antioxidant, hypolipidemic, hypoglycemic, and appetite-regulating effects of three major fractions of chicory root, i.e., inulin, chlorogenic acid, and sesquiterpene lactones, in a mouse model. Inulin was the most involved in the impact, accounting for 83% of the recorded responses [26]. Additionally, many studies have also highlighted the anticancer potential of chicory, with cytotoxicity against a range of cancer cells observed in vitro and in vivo [3]. Unfortunately, most previous research did not specify which species, cultivars, or varieties of *C. intybus* were analyzed. However, it might be assumed that the main focus was on the roots of industrial chicory and witloof chicons, as these parts are commonly used [12]. Therefore, the phytochemical and biological potential of the roots of *C. intybus* var. *foliosum* is less explored and requires further investigation.

The nutritional value and potential therapeutic properties of chicory make it of growing interest in the food and pharmaceutical industry. Chicory root extract for industrial application can be prepared by various methods, such as maceration, acid or enzyme-assisted extraction, reflux extraction, percolation, and cross-flow extraction [9,28,29]. Before extraction, chicory roots often undergo preparatory processing. All these steps may significantly influence the yield, chemical profile, and biological effects of the extracts [30,31].

Therefore, this study focuses on evaluating and comparing the phytochemical potential of the witloof variety (*C. intybus* var. *foliosum*) of chicory cultivated in Poland, and the cytotoxicity of root extracts obtained through three different extraction methods: pectinase-assisted, pressure-assisted, and a combination of both. The content of inulin, total content of polyphenols, and total caffeic acid derivatives were measured by the Layne–Eynon, Folin–Ciocalteu, and UHPLC-PDA methods, respectively. Cytotoxicity of the extracts and their primary component—inulin—was tested in vitro using the L929 cell line and the MTT test. The study formed the basis for verifying the safety, quality assessment, and standardization of the plant material and extracts, which is essential for planning future in-depth research on their pharmacology and health-promoting properties as functional ingredients.

## 2. Materials and Methods

### 2.1. Materials and Reagents

Carrez I ([K_4_(Fe(CN)_6_] × 3H_2_O), Carrez II (ZnSO_4_ × 7H_2_O), concentrated hydrochloric acid d = 1.19 g/cm^3^, methylene blue, 20% aqueous sodium hydroxide solution, Fehling I (CuSO_4_), Fehling II (C_4_H_4_O_6_KNa. 4H_2_O + 100 g NaOH/l H_2_O), Folin–Ciocalteu reagent, pectinase, and saturated sodium carbonate solution were purchased from ChemPure (Piekary Śląskie, Poland).

Triton X-100, low density polyethylene, inulin, paracetamol, and L929 cell line (mouse fibroblasts) were purchased from Sigma-Aldrich (Seelze, Germany/St. Louis, MO, USA).

Medium containing Earle’s balanced salt solution, with phenol red, Fetal calf serum, Trypsin/vermic acid solution, PBS solution: phosphate buffered saline without Ca^2+^ and Mg^2+^, Penicillin/streptomycin solution: 10,000-unit solution (10 mg/mL), NEAA: Solution MEM non-essential amino-acids, sodium pyruvate solution (100 mM), L-glutamine solution (200 mM) was purchased from Biological Industries (Beit Haemek, Izrael).

MTT: 3-(4,5-dimethylthiazol-2-yl)-2,5-diphenyltetrazolium bromide and isopropanol were purchased from Serva (Heidelberg, Germany).

### 2.2. Preparation of Chicory Root Extracts

**Figure 2:**
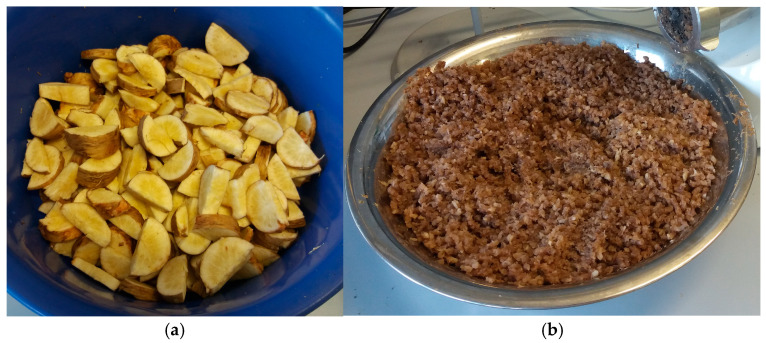
Root of *Cichorium intybus* L. after washing: (**a**) cutting and (**b**) ground root.

Three chicory root preparations were obtained using three different extraction methods (Methods 1–3). The plant material (chicory root of the witloof variety *C. intybus* L. var. *foliosum*) was supplied by a local chicory grower after harvesting and processing including cutting off the leafy heads (Bakor Ltd., Skierniewice, Poland). For extraction, the raw roots were washed with water and ground into pieces (Figure 2) using a grinding machine with sieve mesh diameter of 4 mm.

In Method 1 (pectinase-assisted extraction), 8 L distilled water was added to 5 kg of ground chicory root. Then, the pH of the mixture was adjusted to 4.0 using 0.1 mol/L HCl, and 0.5 mL of the 0.1% pectinase solution in water was added. The temperature was maintained at 50 °C for six hours due to the optimal action of the enzyme in the range of 35–60 °C. With the use of pectinase, extraction can be carried out under milder conditions (lower temperature), which reduces thermal degradation of bioactive compounds. The use of the enzyme has been shown in the literature to increase the extractability of bioactive compounds from chicory roots [32]. In Method 2, the mixture of chicory and water was prepared in the same ratio as in Method 1 (without acidification) and boiled under pressure of 0.2 MPa using a pressure cooker for 30 min. In Method 3, pectinase was added to an acidified solution of ground chicory root and distilled water as in Method 1, and the temperature was maintained at 50 °C for six hours; following this, the solution was boiled in a pressure cooker under pressure as in Method 2. All three extracts were filtered by a Büchner funnel [33] and lyophilized using a Christ Delta 1–24 LSC freeze-dryer (Christ, Osterode am Harz, Germany) and the following conditions: freezing the product at −48 °C for 24 h, basal drying lasting ~20 h at a shelf temperature of 30 °C and a pressure of 0.42 mbar, and re-drying lasting ~4 h at a shelf temperature of 40 °C and a pressure of 0.024 mbar. The lyophilized extracts were stored at −25 °C before further analyses [32].

### 2.3. Determination of Inulin Content by Lane and Eynon Method

The tested chicory root preparations were weighed and dissolved in distilled water. The extracts thus obtained were brought to room temperature, and then 50 mL of the solution was accurately measured into a 250 mL conical flask using a glass pipette. A stock solution was prepared, and protein was removed from the test sample (deproteinization) using Carrez I and II. The sugar content was determined by copper reduction; next, the mixture was inverted using concentrated hydrochloric acid, and the sugar content was determined again after inversion. The sugar contents were determined by Lane and Eynon titration using Fehling’s solution as described in AOAC (2019) method no. 925.35 [30,34].

### 2.4. Determination of Total Phenolic Content by the Folin–Ciocalteu Method

The content of total polyphenolic compounds was determined using a modified Folin–Ciocalteu (F-C) method optimized for chicory samples, as described by Sinkowic et al. [35]. While this method differs from the AOAC standard procedure, it was selected due to its demonstrated effectiveness in analyzing chicory-specific polyphenols, particularly chlorogenic acid, which is the predominant polyphenol in chicory. The reaction mixture contained 50 µL of 1% chicory extract solution, 3.85 mL of distilled water, 100 µL of FC reagent, and 1 mL of saturated Na_2_CO_3_ solution. The mixture was incubated in the dark at 25 °C for 30 min. After incubation, absorbance was measured at 725 nm using a T60 spectrophotometer (PG Instruments, Lutterworth, UK). The concentration of phenolic compounds in the extracts was calculated using a standard curve of chlorogenic acid and expressed in mg chlorogenic acid equivalents/100 g dw.

### 2.5. UHPLC-PDA Quantitation of Caffeic Acid Derivatives

The quantitative UHPLC-PDA analysis of the chicory root extracts was performed using Nexera X3 (Shimadzu, Kioto, Japan) system equipped with the LC-40 pump, the autosampler SIL-40C, a thermostat CTO-40C, and the photo diode array detector SPD-M30A. Separations were carried out on the ARION Polar C18 (2.2 µm, 2.1 × 100 mm) column at 35°C. A mobile phase consisted of the solvent A (acetonitrile), and the solvent B (water:orthophosphoric acid, 99.5:0.5 *v*/*v*), with the following elution profile: 0–3 min, 3% A; 3–26 min, 3–40% A; 26–30 min, 40–90% A; 30–35 min, 90% A; 35–36 min, 90–3% A; 36–46 min, 3% A (equilibration). The flow rate was 0.3 mL/min, and the injection volume 1 µL. Before injections, sample solutions (13 mg/mL) of the extracts in acetonitrile–water (7:3, *v*/*v*) were filtered through a PTFE syringe filter (25 mm, 0.2 µm, VWR, Randor, PA, USA). UV-Vis spectra were recorded over the range of 200–600 nm. The peaks presenting spectra typical for caffeic acid derivatives were divided into the groups of monocaffeoyl (monocaffeoylquinic acids/monocaffeolytartaric acids, MCA) and dicaffeoyl derivatives (dicaffeolyquinic acids/dicaffeoyltartaric acids, DCA) by comparing their retention times (t_R_) with reference standards, based on our previous studies [36]. Eventually, peaks eluting before t_R_ = 15 min were assumed as MCA, and those eluting after t_R_ = 15 min as DCA. For quantitation purposes, the detection wavelength was set at 325 nm, and the analytes were quantified as equivalents of HPLC-pure external standards (Sigma-Aldrich, Seelze, Germany/St. Louis, MO, USA): chlorogenic acid for MCA and cynarin for DCA.

### 2.6. In Vitro MTT Cytotoxicity Test of Chicory Extracts

The MTT test was conducted in accordance with the EN ISO 10993–5:2009 standard [37]. The MTT test is a colorimetric assay that determines cell viability by measuring the activity of energy transformations occurring in mitochondria. Enzymatically active mitochondria reduce the water-soluble yellow tetrazolium salt (MTT-3-(4,5-dimethylthiazol-2-yl)-2,5-diphenyltetrazolium bromide) to blue–violet insoluble formazan. The number of living cells is proportional to the intensity of formazan.

The L929 cells (mouse fibroblasts) were seeded in a 96-well plate at a density of 10^4^ cells per well and then cultured for 24 hours at 37 °C and 5% CO_2_. The medium was then removed, and the cells exposed to 100 μL of the compound/extract solutions of various concentrations (final DMSO concentration in the culture medium was below 0.2%) or only the culture medium (blank control). Pure DMSO was used as a positive control. The cells with the solutions were incubated for 24 hours. The medium was then removed and 50 μL of the MTT solution was added to each well and incubated in the dark for two hours at 30 °C. The MTT solution was then carefully removed using a 12 × 200µL serial pipette and 100 μL of DMSO was added. The plates were kept at room temperature for 10 min.

Following this, 5 μL of Sorensen buffer was added to each well and placed in a microplate reader (Synergy H1, BioTek, Winooski, VT, USA). The plate was rocked, and the absorbance measured at 570 nm. Cell viability was expressed as a percentage of control values (blank). The reduction in viability, compared to the control group, was calculated as follows:

$$\mathrm{Survival}\mathrm{rate}\;(\%)\;=\;\frac{100\%\cdot Ae}{Ak}$$

where Ae is the mean absorbance value of at least three repetitions of the 100% extract of the test sample/positive control/negative control, and its subsequent dilutions; Ak is the mean absorbance value of blank control, not treated by the extracts or test compounds. If viability is reduced to <70%, the test sample is assumed cytotoxic. The 50% dilution of the test sample should have at least the same or higher viability than the 100% sample, otherwise the test should be repeated [37,38,39].

### 2.7. Statistics

The analysis for the physicochemical tests was carried out using the STATISTICA 13.1 software from StatSoft Inc., Tulsa, OK, USA, with a significance level of *p* < 0.05.

Statistical analysis for cytotoxicity results was performed using GraphPad Prism v8 (GraphPad Software, San Diego, CA, USA). The normality of distribution was determined with the Shapiro–Wilk test. Since data passed the normality test, the ordinary one-way ANOVA was used alongside the post hoc Sidak’s multiple comparison test. Results with a *p*-value less than 0.05 were considered statistically significant.

## 3. Results and Discussion

### 3.1. Inulin and Polyphenol Content

The results of the inulin and polyphenol analyses are shown in Table 1. The highest inulin content was obtained by the combined method of pectinase-assisted and pressure-assisted extraction, and the lowest in extracts obtained by the enzymatic treatment only (*p* < 0.05). This may be due to the synergistic effect of high temperature and enzymatic activity, which, when combined, more effectively destroy the cell structure and release bioactive compounds. Heating at elevated pressure could enhance the disintegration of the cell wall, facilitating enzymatic access to substrates and improving the extraction of polysaccharides. Alternatively, the enzymatic method could be less effective at low temperatures, resulting in lower inulin extraction efficiency [40,41,42].

Previous studies indicate that combined methods give higher concentrations of bioactive compounds than enzymatic methods alone, possibly due to the effect of temperature [43,44]. For example, optimal conditions for inulin extraction are 60–80 °C, which is consistent with the need for higher temperatures to maximize the enzyme efficiency [40]. Nevertheless, while the differences between the individual methods 1–3 are statistically significant, they are relatively low and insufficient to clearly indicate the most effective one based on the obtained results.

**Figure 3:**
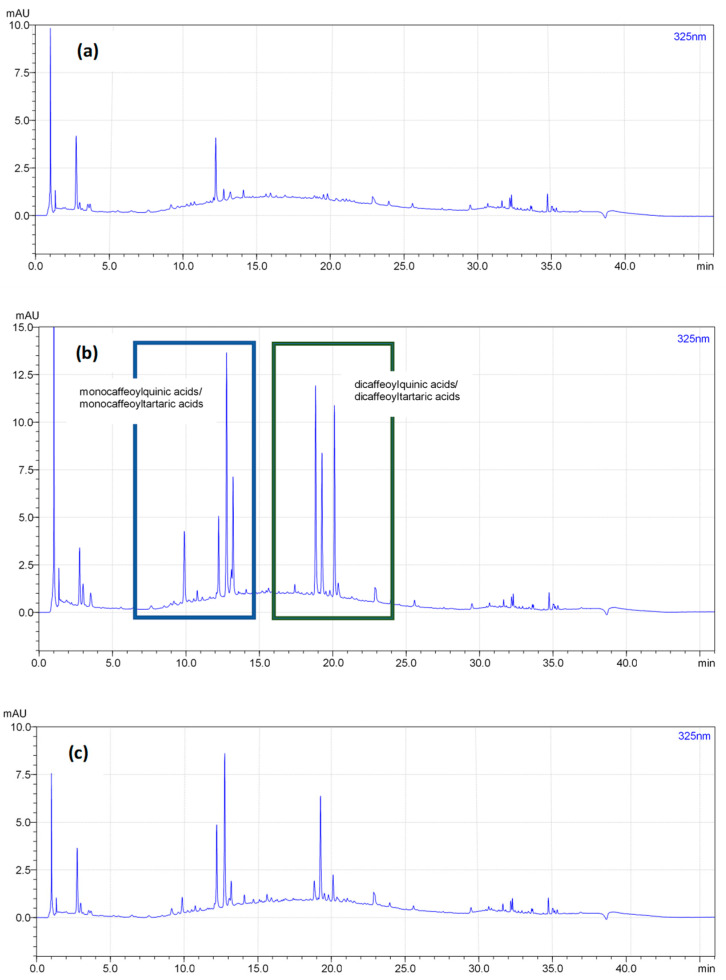
Representative UHPLC-PDA chromatograms at 325 nm of the chicory root extracts obtained using three methods: (**a**) pectinase-assisted extraction (Method 1); (**b**) pressure-assisted extraction (Method 2); (**c**) combined pectinase-assisted and pressure-assisted extraction (Method 3). The boxes drawn in chart (**b**) show the elution range of monocaffeoyl and dicaffeoyl derivatives.

The polyphenolic fraction was also analyzed in the extracts to get a more insightful view into the impact of the extraction technique on the recovery of active constituents of chicory roots. A similar tendency to that observed for inulin was noted for the total content of polyphenols (TPC level) measured by the Folin–Ciocalteu method (Table 1); however, the differences between the extraction efficiencies of three tested extraction methods were not statistically different (*p* > 0.05). In contrast, total caffeic acid derivatives (measured by the UHPLC-PDA) varied significantly between the extracts (Table 1). The highest contents of monocaffeoyl and dicaffeoyl derivatives were revealed for the pressure-assisted extraction, followed by the combined extraction method and pectinase-assisted one. As illustrated in Figure 3, the use of pectinase also decreased the complexity of the phenolic fraction in the extracts, which resulted in a lower number of phenolic metabolites (peaks) that could have been detected and quantified in the extracts. This decrease is probably caused by the hydrolysis of caffeic acid esters in an acidic environment required for the optimum enzyme action.

Previous studies indicated that caffeic acid derivatives, including pseudodepsides of quinic and tartaric acids, are primary polyphenols of all varieties of chicory roots, both wild and cultivated [12,13,15]. The phenolic acid fraction of the roots is mainly composed of mono- and dicaffeoylquinic acids, such as isomers of chlorogenic acid (5-*O*-caffeoylquinic acid) and cynarin (1,3-di-*O*-caffeoylquinic acid), which are accompanied by tartaric acid analogues, e.g., chicoric acid. The content of monocaffeoylquinic acids in the root of industrial chicory (0.3% dm) usually exceeds the levels of dicaffeoylquinic acids (0.2%) [15]. Our results for the pressure-assisted extraction (without acidification) suggest that in the roots of the witloof variety, this difference may be lower as both types of these compounds occurred in the root extracts at similar levels, i.e., 93–94 mg/100 g dm (Table 1). In contrast, the recovery-reducing effect of enzyme treatment was more potent for dicaffeoyl derivatives, resulting in their relatively lower levels compared to monocaffeoyl esters in the extract obtained with the combined extraction procedure, i.e., 38.5 versus 54.6 mg/100 g dm (Method 3). All these data confirm that some degradation processes, such as partial hydrolysis of ester bonds, may take place during enzymatic extraction due to an acidic pH. Partial degradation of chicory hydroxycinnamoyl esters and sesquiterpene lactones after treatment with various enzymes, including pectinase, has also been recently reported for root by-products of both witloof and industrial varieties [45]. In addition, it has been demonstrated that the TPC levels in chicory roots are relatively stable during enzyme treatment [46]. These findings follow our results and suggest that some non-phenolic, hydrolysis-resistant compounds might be co-responsible for the extract responses in the Folin–Ciocalteu reaction. Considering the redox mechanism of this reaction, the TPC levels may be assumed as an indirect measure of the antioxidant potency of the chicory root extracts.

Although the pressure-assisted and combined extraction methods showed higher extraction efficiency for both inulin and polyphenols, the enzymatic method alone may still be advantageous for specific applications, especially where selectivity for inulin is required. This highlights the importance of selecting a method based on the expected results [47]. Previous studies have found the inulin content obtained by melting the frozen aqueous extract prepared from 100 g of dried chicory roots to be 18.1 g for oven drying and 35.5 g for sun drying [48]. In other studies, inulin percentages in the chicory extract ranged from 11.67% to 65.60% [49].

The high value of the tested witloof roots as a source of inulin is worth highlighting as significantly lower levels of this polysaccharide are available from other plants. For instance, its content in garlic is about 18% of fresh weight [50], and ranges from 8.16% to 13.46% of fresh weight in Jerusalem artichoke [51].

### 3.2. MTT Cytotoxicity Studies

The cytotoxicity findings (IC_50_) for the extracts from the three methods, pure inulin, and the reference drug paracetamol are shown in Table 2 and Table 3.

The IC_50_ values for individual chicory extracts and comparators ranged from 3.02 to 19.77 µg/mL. Among the extracts, the preparation obtained by the enzymatic method achieved the highest mean IC_50_ value of 7.31 µg/mL (SD: 1.0, RSD: 13.67%), indicating the lowest cytotoxicity. In contrast, the combined method yielded the most cytotoxic sample with an IC_50_ value of 4.72 µg/mL (SD: 0.58, RSD: 12.22%); however, the difference in cytotoxicity between this and pressure-assisted extraction is relatively small. For comparison, the mean IC_50_ of the analgesic drug (paracetamol) was 3.02 µg/mL (SD: 0.22, RSD: 7.19%), i.e., it revealed significantly higher cytotoxicity than all three chicory extracts and inulin. In contrast, with the mean IC_50_ value of 19.77 µg/mL (SD: 2.40, RSD: 12.15%), the control inulin demonstrated the lowest cytotoxicity among all tested samples and pure compounds.

The differences in cytotoxicity observed for chicory extracts obtained with various methods followed the previous reports, indicating that IC_50_ values may differ even for the same substance, because they depend on the type of biological activity tested [52], the degree of polymerization of the compound, the origin of the substance, the purity of the preparation, or even the method of calculating the final value [53].

For instance, in the previous studies on chicory extracts, the IC_50_ values in the MTT test differed significantly depending on the part of the plant and the extraction method used. Studies have also indicated that methanolic extracts generally have a stronger cytotoxic effect than aqueous extracts. For chicory stem extracts, IC_50_ values were as follows: methanol extract—0.64 mg/mL, aqueous extract—2.44 mg/mL, and, for chicory leaf extracts: 0.69 mg/mL for methanol extract and 2.58 mg/mL for the aqueous one. Methanol extracts consistently showed lower IC_50_ values, i.e., higher cytotoxicity, than aqueous extracts, regardless of the plant part [54].

Few studies have assessed the IC_50_ of inulin in the MTT assay. One study found paclitaxel-loaded inulin stearate micro-rods to have an IC_50_ value of 3.1 μM in the HeLa cell line; however, the potent cytostatic drug contributed chiefly to the obtained results [55]. Regarding inulin-like substances, such as beta-glucan, the IC_50_ measured by the MTT assay was as low as 200 μg/mL [56]. Studies indicate that paracetamol showed aromatase-inhibitory activity with IC_50_ values of 14.7 × 10^−5^ M and 57.6 × 10^−5^ M, respectively [57]. Paracetamol is one of the most popular and widely used antipyretics and painkillers worldwide. It is available without a prescription in single- and multi-ingredient preparations. Its mechanism of action is complex and involves both central effects, viz. COX, serotonergic descending neuronal pathway, L-arginine/NO pathway, cannabinoid system, and peripheral processes, including inhibition of COX antinociception and the “redox” mechanism. However, it should be borne in mind that, despite its many advantages, regular use at doses over 4 g/day can result in, among others, liver damage or even death [58].

**Figure 4:**
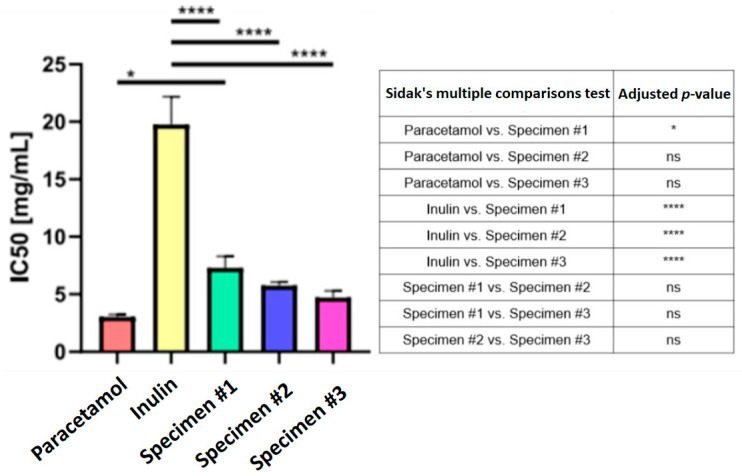
IC_50_ for 3 chicory extracts (Specimen #1, Specimen #2, Specimen #3), paracetamol, and inulin. Statistical significance was assessed using GraphPad Prism v8 (GraphPad Software, San Diego, CA, USA) with a post hoc analysis. **** *p* < 0.0001, *** *p* < 0.001, ** *p* < 0.01, and * *p* < 0.05 were considered as significant.

A detailed analysis of the cytotoxicity test performed in the present study is given in Figure 4. Significant differences (*p* < 0.0001) were noted between all tested extracts (Specimen #1—Method 1: enzyme-assisted extraction, Specimen #2—Method 2: pressure-assisted extraction, Specimen #3—Method 3: enzyme + pressure-assisted extraction), inulin, and paracetamol. Results may suggest that the presence of inulin in the extracts has only a limited effect on the cytotoxic properties of the tested samples. The lack of statistically significant differences between the IC_50_ values for paracetamol and the extracts obtained by the pressure-assisted and combined extraction methods suggests their similar cytotoxic effects on the L929 cell line (mouse fibroblasts). As such, despite their differences in chemical composition, these extracts might mimic the cytotoxic effects observed for paracetamol. In addition, the lack of statistical differences between the individual extracts indicates that the extract preparation method has little impact on their cytotoxicity. This result suggests that since modifications to the processing do not lead to significant changes in cytotoxic activity, selecting the most effective and economical extraction method should be based on the phytochemical value of the extracts and recovery of the active compounds.

## 4. Conclusions

The article compared three extraction methods (enzymatic and pressure-assisted extraction, and both combined) of witloof roots, with regard to the polyphenol and inulin extraction efficiency, as well as the potential cytotoxicity of the obtained extracts. Inulin content ranged from 43.88 to 50.95 g/100 g, the total polyphenolic content was between 816.7 and 906.4 mg/100 g, and total phenolic acids ranged from 11.50 to 187.1 mg/100 dm. As for the cytotoxicity, the IC_50_ value ranged from 4.72 to 7.31 mg/mL for chicory extracts, 3.02 for paracetamol, and 19.77 for inulin. The results justify further studies of the medicinal and functional properties of the roots of *C. intybus* L. var*. foliosum*.

While combining enzymatic action with elevated pressure treatment allows the highest recovery of inulin and total polyphenols, the relatively high cytotoxicity of this extract may limit its use in some contexts, such as repeated administration of concentrated preparations for medicinal purposes. However, the synergistic combination of enzymes and elevated pressure may be valuable in other applications where cytotoxicity is not a key criterion.

Although the enzyme-assisted method is the safest in terms of cytotoxicity, it is not adequately effective in terms of inulin and polyphenol extraction efficiency. The lower temperature limits degradation of cellular structures, while the acidic pH stimulates hydrolytic cleavage of caffeic acid esters, which results in a lower content of bioactive compounds. Therefore, this method is more suitable in cases where the priority is to minimize toxicity, and the content of bioactive compounds is less important.

The method based only on pressure-assisted extraction represents a compromise between extraction efficiency and cytotoxicity, and is the optimal choice for further studies. It offers similar results to the combined method regarding inulin and total polyphenolic content, while increasing the levels of caffeic acid derivatives and maintaining lower toxicity. Additionally, this method is economical and less complicated, favoring its potential implementation in industrial processes.

The aqueous extracts are more suitable than methanolic ones, as confirmed by literature data indicating that the aqueous extracts have lower IC_50_ properties. Therefore, aqueous extracts seem to be a desirable choice in future research on the safety and effectiveness of *Cichorium intybus* L. preparations.

Several potential directions for future investigations can be identified. These include further optimizing extraction methods to obtain extracts with desired cytotoxicity and safety, and identifying the mechanisms underpinning the differences in cytotoxicity. They could also explore the influence of extract composition on biological activity. By using chicory extracts with lower cytotoxicity than the reference drug paracetamol, it will be possible to explore their potential therapeutic applications. In future studies, we plan to focus on the analgesic properties of chicory roots.

## Figures and Tables

**Table 1:** Inulin and polyphenol content in chicory extracts obtained by three extraction methods.

Constituent	Enzyme-Assisted Extraction (Method 1)	Pressure-Assisted Extraction (Method 2)	Enzyme + Pressure-Assisted Extraction (Method 3)
**Inulin** (g/100 g dm)	43.88 ± 0.98 c	47.34 ± 1.47 b	50.95 ± 0.69 a
**TPC** (mg/100 g dm)	816.7 ± 56.4 b	881.2 ± 49.6 b	906.4 ± 14.1 b
**MCA** (mg/100 g dm)	11.46 ± 0.08 c	94.08 ± 2.12 a	54.57 ± 2.67 b
**DCA** (mg/100 g dm)	<LOQ	92.99 ± 0.88 a	38.49 ± 1.57 b
**TCA** (mg/100 g dm)	11.46 ± 0.08 c	187.1 ± 3.01 a	93.07 ± 4.23 b

Results are presented as means ± SD (n = 6). Different superscript letters in one row indicate significant differences (*p* < 0.05) in Tukey’s HSD test. Abbreviations: dm, dry matter; LOQ, limit of quantitation; TPC, total phenolic content, determined by Folin–Ciocalteau method and expressed in chlorogenic acid equivalents; MCA, total content of monocaffeoyl derivatives, determined by UHPLC-PDA and calculated as chlorogenic acid; DCA, total content of dicaffeoyl derivatives, determined by UHPLC-PDA and calculated as cynarin; TCA, total content of caffeic acid derivatives (sum of MCA and DCA).

**Table 2:** Cytotoxicity test results for chicory extracts obtained by three extraction methods.

Enzyme-Assisted Extraction (Method 1)		Pressure-Assisted Extraction (Method 2)		Enzyme + Pressure-Assisted Extraction (Method 3)	
IC_50_ (mg/mL)		IC_50_ (mg/mL)		IC_50_ (mg/mL)	
mean	7.31	mean	5.76	mean	4.72
SD	1.00	SD	0.32	SD	0.58
RSD (%)	13.67	RSD	5.51	RSD	12.22

Results are presented as means ± SD. SD—standard deviation, RSD—relative standard deviation (coefficient of variation).

**Table 3:** Cytotoxicity test results for a standard painkiller (paracetamol) and inulin.

Paracetamol		Inulin	
IC_50_ (mg/mL)		IC_50_ (mg/mL)	
mean	3.02	mean	19.77
SD	0.22	SD	2.40
RSD (%)	7.19	RSD	12.15

Results are presented as means ± SD. SD—standard deviation, RSD—relative standard deviation (coefficient of variation).

## Data Availability

The data presented in this study are available on request from the corresponding authors.
